# Improving Nitrogen Use Efficiency in Aerobic Rice Based on Insights Into the Ecophysiology of Archaeal and Bacterial Ammonia Oxidizers

**DOI:** 10.3389/fpls.2022.913204

**Published:** 2022-06-13

**Authors:** Muhammad Shahbaz Farooq, Muhammad Uzair, Zubaira Maqbool, Sajid Fiaz, Muhammad Yousuf, Seung Hwan Yang, Muhammad Ramzan Khan

**Affiliations:** ^1^Institute of Environment and Sustainable Development in Agriculture, Chinese Academy of Agricultural Sciences, Beijing, China; ^2^National Institute for Genomics and Advanced Biotechnology, Islamabad, Pakistan; ^3^Institute of Soil Science, Pir Mehr Ali Shah-Arid Agriculture University, Rawalpindi, Pakistan; ^4^Department of Plant Breeding and Genetics, The University of Haripur, Haripur, Pakistan; ^5^Pakistan Agricultural Research Council, Islamabad, Pakistan; ^6^Department of Biotechnology, Chonnam National University, Yeosu, South Korea

**Keywords:** aerobic rice system, N-cycle, ammonia-oxidizers, nitrification-inhibitors, agronomic adjustive measures

## Abstract

The abundance and structural composition of nitrogen (N) transformation-related microbial communities under certain environmental conditions provide sufficient information about N cycle under different soil conditions. This study aims to explore the major challenge of low N use efficiency (NUE) and N dynamics in aerobic rice systems and reveal the agronomic-adjustive measures to increase NUE through insights into the ecophysiology of ammonia oxidizers. Water-saving practices, like alternate wetting and drying (AWD), dry direct seeded rice (DDSR), wet direct seeding, and saturated soil culture (SSC), have been evaluated in lowland rice; however, only few studies have been conducted on N dynamics in aerobic rice systems. Biological ammonia oxidation is majorly conducted by two types of microorganisms, ammonia-oxidizing archaea (AOA) and ammonia-oxidizing bacteria (AOB). This review focuses on how diversified are ammonia oxidizers (AOA and AOB), whose factors affect their activities and abundance under different soil conditions. It summarizes findings on pathways of N cycle, rationalize recent research on ammonia oxidizers in N-cycle, and thereby suggests adjustive agronomic measures to reduce N losses. This review also suggests that variations in soil properties significantly impact the structural composition and abundance of ammonia oxidizers. Nitrification inhibitors (NIs) especially nitrapyrin, reduce the nitrification rate and inhibit the abundance of bacterial *amoA* without impacting archaeal *amoA*. In contrast, some NIs confine the hydrolysis of synthetic N and, therefore, keep low NH_4_^+^-N concentrations that exhibit no or very slight impact on ammonia oxidizers. Variations in soil properties are more influential in the community structure and abundance of ammonia oxidizers than application of synthetic N fertilizers and NIs. Biological nitrification inhibitors (BNIs) are natural bioactive compounds released from roots of certain plant species, such as sorghum, and could be commercialized to suppress the capacity of nitrifying soil microbes. Mixed application of synthetic and organic N fertilizers enhances NUE and plant N-uptake by reducing ammonia N losses. High salt concentration promotes community abundance while limiting the diversity of AOB and vice versa for AOA, whereas AOA have lower rate for potential nitrification than AOB, and denitrification accounts for higher N_2_ production. Archaeal abundance, diversity, and structural composition change along an elevation gradient and mainly depend on various soil factors, such as soil saturation, availability of NH_4_^+^, and organic matter contents. Microbial abundance and structural analyses revealed that the structural composition of AOA was not highly responsive to changes in soil conditions or N amendment. Further studies are suggested to cultivate AOA and AOB in controlled-environment experiments to understand the mechanisms of AOA and AOB under different conditions. Together, this evaluation will better facilitate the projections and interpretations of ammonia oxidizer community structural composition with provision of a strong basis to establish robust testable hypotheses on the competitiveness between AOB and AOA. Moreover, after this evaluation, managing soils agronomically for potential utilization of metabolic functions of ammonia oxidizers would be easier.

## Introduction

Human-based activities and climate change have fiercely shifted the global nitrogen (N)-cycle with increased amount of reactive N in the biosphere. Shifts in N cycle have reasonably resulted from interactions of N pools with crops and soil under changed climatic conditions, which are subjected to global warming and extreme stress events (Fowler et al., 2013). Enhanced use of synthetic and industrially manufactured N fertilizers in terrestrial habitats, and N_2_ fixation due to interactions of symbiotic microorganisms and plants have exceeded the natural N-inputs added to anthropic N inputs (Fowler et al., 2013; Zhang et al., 2019a,b). The N use efficiency (NUE) of synthetic N fertilizers in agriculture remain fairly low; ordinarily, 50% or even less of the N fertilizer applied is taken up by plants (Millar et al., 2014). Conventional rice systems need several transformational approaches that reduce water consumption to balance the economic, environmental, and production status under future threats of scarce availability of freshwater resources. Transformational modifications (aerobic rice systems) in conventional rice sustain yield but at the cost of higher N loss. Therefore, comprehensive understanding and comprehension of recent advances in the N cycling of aerobic rice systems under irrigated conditions is necessary to intensify a sustainable rice yield while reducing N loss and environmental hazards (Awan et al., 2014; Javed et al., 2021; Uzair et al., 2022). The ecophysiology, abundance, and activities of soil microbes involved in N cycling processes change under different crop production systems. Therefore, explication of diversity and functions of soil microbes that are dominant in N cycling require a comprehensive understanding of the ecophysiology of key ammonia-oxidizing microorganisms and thereby suggest agronomic adjustive measures to enhance NUE (Wang et al., 2019). Classification and evaluation of essential considerations like N-fertilizer management (Snyder, 2016) especially for nitrification (explanation in later parts) may enhance NUE while reducing greenhouse gas (GHG) emission and N loss and the transport of reactive N, avoiding environmental pollution (Shiozaki et al., 2016; Smith et al., 2016). Ammonia-oxidizing bacteria (AOB) and ammonia-oxidizing archaea (AOA) are among the dominant microbes playing crucial roles in N cycle (Sauder et al., 2017; Wang H. et al., 2017). The current knowledge of the ecophysiology of vital ammonia-oxidizing microbes has been primarily based on pure isolates, but studies under natural conditions are rare (Tourna et al., 2011). Therefore, the present study is aimed to provide a deep insight into the ecophysiology of major soil ammonia oxidizers (AOA and AOB); understanding the major pathways of ammonia oxidation and mechanisms of how active ammonia oxidizers interactively impacts N cycling and plant N-uptake, thereby recommending adjustive measures to limit the N-losses.

The first part of this article reviews the importance of bringing transformations (aerobic rice systems) in conventional rice systems and how N-cycle occurs under aerobic rice systems with emphasis on the importance of reducing N loss. Then, it moves toward elaboration of important reactions in N-cycle viz. ammonification, nitrification, denitrification, and volatilization and leaching. Later, it expands the understanding toward the ecophysiology, abundance, diversification, and activities of microbial communities in aerobic rice systems and how their growth and activities are affected by various environmental factors like soil temperature, pH, NH_4_^+^ availability, soil dissolved oxygen, and moisture content. The later parts of the article review the pathways of action of AOA and AOB, and thereby recommend several soil, crop, and fertilizer adjustive management strategies to optimize the abundance and activities of ammonia oxidizers.

### Climate Change and Rice Production

Concrete bodies of evidence have revealed that earth mean surface temperature has been warming since the 19th century. Based on the temperature change evidence observed over the last century, it has been observed that global average temperature has increased by 1.09°C since the middle of the 19th century according to the IPCC AR6 WGI report, and that most of the temperature change was noticed in the last three decades of the 19th century (IPCC, 2015). Climate change occurs because of three major factors: natural factors, human-induced factors, such as GHGs and methane (CH_4_) emission, and land use shifts. Human-induced factors enhanced the levels of atmospheric carbon dioxide (CO_2_) from 284 ppm in 1832 to 410 ppm in 2013 (Hoffman et al., 2014), which ultimately increased the temperature and caused global warming. It is likely that climate change will produce severe changes in temperature, precipitation frequency and patterns, and extreme stress events. Undergoing this projection, it is anticipated that global warming patterns will not be even across the globe, and that arid and oceanic parts will be under more threats (Solomon et al., 2007). Meanwhile, it has also been revealed that average earth temperature will rise more slowly than projected with climatic models because of higher absorption of CO_2_ by oceans (Balmaseda et al., 2013). Moreover, due to unevenness and irregular climatic changes, there are uncertainties regarding precipitation patterns, intensity, and frequency, specifically over tropical regions, due the inefficacy of models to demonstrate an accurate hydrological cycle (Lorenz et al., 2012).

Climate change has threatened global rice production especially in major rice-cultivating Asian countries. There is an important linkage between rice production and climate change as the former contributes to global climate warming because of higher rate of CH_4_ and other GHG emission, while rice systems are getting impacted by climate change. Rice production is required to be increased by 40% by the end of 2030 because of scarcity of resources to meet the world’s growing population staple food demands (FAO, 2016), which may exacerbate the problem of environmental hazards because of increased CH_4_ and GHG emissions. Globally, China and India are the most densely populated countries sharing 20 and 28.5% of total global rice area, respectively (Hwalla et al., 2016) and, therefore, have a large share in CH_4_ emission. Comparing with the area division, 90 and 46% of rice production in China and India, respective, is irrigated (Jain et al., 2000; Pathak et al., 2005). Rice, during flooding conditions, acts as one of the main sources of CH_4_ (Oelbermann, 2014). Various transformed rice production practices, including aerobic rice systems, have been introduced, are resource-use efficient, and can sustain grain yield but may create environmental hazards (N oxide emission) because of higher N loss although reduce the emission of CH_4_.

The emission rate of GHGs from rice soil is greatly dependent on soil organic matter (OM) status, land use changes, cropping intensity, irrigation management practices, soil microbial abundance and their functioning, soil properties, and environmental variables. Normally, GHG emission, especially CH_4_, is higher in flooded rice systems. However, the transformation of conventional rice systems to aerobic rice systems may fulfill future food demands by sustaining grain yield under scarcity of inputs but at the cost of higher N loss, such as nitrous oxide (N_2_O), through coupled nitrification-denitrification processes under an alternate wetting and drying (AWD) irrigation system (Huang and Gerber, 2015). Therefore, understanding the shifts in microbial abundance and activities and pathways of N-cycle under aerobic rice systems is necessary to reduce N loss undergoing several agronomic management measures enhancing NUE, thereby reducing environmental hazards.

### Need for Transformation of Conventional Rice Systems to Aerobic Rice Systems

Scarcity of fresh water resources is bringing changes in irrigated rice systems, such as to switch toward a water-use efficient direct-seeded rice system accompanying AWD irrigation management (Joshi et al., 2013). This necessarily requires the identification of the most suitable cultivars with high yield and resource use efficiency features under different nutrient and water management regimes (Nie et al., 2012; Kumar et al., 2022). More than 75% of global rice comes from 79 million ha of irrigated rice areas across the globe, and it has been predicted that 17 million ha of irrigated rice in Asia will experience “physical water scarcity” and that 22 million ha will be subjected to “economic water scarcity” (Tuong and Bouman, 2003). By the end of 2050s, agricultural output must increase by 70% to feed over nine billion people mostly living in urban areas across the globe (FAO, 2017). Considering this challenge, the required increase in agricultural output has to be achieved with diminishing resources of water, land, and energy. Scarcity of resources has driven the focus on improving the general eco-efficiencies of agricultural systems. Eco-efficiency is about achieving more with less, i.e., more agricultural outputs per unit application of inputs, both in terms of quantity and quality (Keating et al., 2010). The overall aim of this concept is to increase the productivity of low-input systems (rainfed upland rice) and the sustainability of high-input systems (irrigated lowland rice) through transformational technologies (aerobic rice systems) (Bouman et al., 2005, 2006). The aerobic rice system is a new resource-use efficient, initiated by the International Rice Research Institute (IRRI) and is an alternative to the conventional rice system for high production under non-flooded, non-puddled, and unsaturated soil conditions. The aerobic rice system is considered as highly receptive to nutrient availability, can be rainfed or irrigated, and occasionally tolerates flooding (Bouman and Tuong, 2001), and this system has been successfully adopted in countries like China, Brazil, and many other Asian rice-producing countries (Kato and Katsura, 2014). Therefore, it can be an alternative to the conventional rice system with typically high resource-use efficiencies and profitability for farmers (Kadiyala et al., 2012; Djaman et al., 2018).

### N Use Dynamics Under Aerobic Rice Systems

Currently, global rice systems are suffering from higher N loss next to water, which shares key roles in vegetative growth, and grain, and reduces evaporative water loss (Jia et al., 2019). Rice grown under aerobic soil conditions utilizes NO_3_^–^-N, which minimizes ammonia (NH_3_) volatilization (Froes de Borja Reis et al., 2018). However, the AWD irrigation management of aerobic rice systems causes decomposition of OM, which is subjected to N loss in the form of NH_3_, and when the soil becomes dry, rapid coupled processes of nitrification-denitrification lead to more losses of N in the form of N_2_O (Sahrawat, 2010). Relative to lowland rice, the abundant form of N in aerobic rice systems is NO_3_^–^, which is subjected to lower NH_3_ volatilization loss, the prevailing form of N loss in lowland rice (Sahrawat, 2010, 2012). Intermittent soil wetting and drying lead to high ammonification of N from decomposition of OM under dry conditions, followed by NH_3_ loss during flooding conditions. Then, the soil becomes dry; again, ammonification followed by nitrification-denitrification processes lead to production of N_2_ and N-oxides (Cui et al., 2018). In aerobic rice systems, production response mechanisms in terms of apparent N recovery to total N applied and agronomic N-use efficiency are strongly affected by water supply (Fuhrmann et al., 2018), crop management practices, and microbial activities. Net productivity, N uptake, and biomass production can be enhanced with more N supply but with higher N loss and GHG emission, because the quantity of N available was higher than its uptake (Zhou et al., 2017; Huang et al., 2022). Therefore, for widespread adaptation of aerobic rice systems, understanding the shifts in N use dynamics under aerobic soil conditions is necessary, thereby reducing N loss and serious environmental hazards through agronomic management (Nie et al., 2009).

### Importance of Increasing N Use Efficiency

Policy experts and scientists involved in climate change issues contend that N_2_O, one of the major GHG and the third ozone depletion agent, demands more focused consideration from agricultural and climate science research collaborations (Kanter et al., 2013). Human-induced activities have significantly impacted agricultural systems by increased use of synthetic inputs and changed the major biogeochemical processes especially N cycling, and contribute to high GHG emission. Agriculture is one of the major sources responsible for contribution in two-thirds of anthropogenic emissions directly or indirectly (Wang et al., 2013). Hence, agricultural and nonagricultural GHG emissions (energy generation sector, industry, and transport) increased the atmospheric concentration of N_2_O from 275 ppb in 1950 to 328 ppb in 2010 (Butler and Montzka, 2015). However, N_2_O is one of the major causes of GHG emission that aggravates climatic changes; its emission is also impacted by climate change. Rise in atmospheric temperature, changes in precipitation patterns and soil water contents, and increase in CO_2_ concentration directly impact N cycling, because N_2_O emission rate is dependent on these factors, several indirect impacts on N cycling because of variations in environmental components, which stimulate changes in CO_2_ concentrations and plant N uptake. The increase in temperature induced by climatic changes increases the rate of decomposition of soil OMs (Zhu et al., 2019; Khan et al., 2021), releasing the more mineral form of N into the soil medium and ultimately ensuring the more availability of N for nitrification and denitrification pathways (Fowler et al., 2015). Functions of soil oxygen levels are nitrification and denitrification that are inversely proportional to overall water contents in the soil. Meanwhile, soil water contents are a function of soil texture, balance between evapotranspiration and precipitation, and topography. Therefore, the impacts of increase in temperature and changes in precipitation patterns could change the overall N cycle through impacts on rates of nitrification and denitrification.

Additionally, it has been evidenced that increase in atmospheric CO_2_ concentrations indirectly impacts N cycle through shifts in N_2_O emission (Zaehle, 2013). Limited crop performance has been observed under N deficiency during elevated CO_2_ conditions, depending on the ability of plant N-uptake. Moreover, elevated CO_2_ can reduce soil N-availability because of probable increase in microbial growth, leading to enhancement of inorganic N in the soil. Under elevated CO_2_ conditions, higher carbon fertilization causes increase in photosynthesis while decreasing transpiration because of increase in plant N uptake, which ultimately decreases N_2_O emission (Venterea et al., 2012; Xu-Ri et al., 2012). The effect of continuity and magnitude of carbon fertilization mainly depends on the access of plants to acquire the available mineral N. Limited N availability is a major issue especially for natural ecosystems where main sources of N inputs are not able to maintain increased plant N demands stimulated by higher carbon fertilization (Zaehle, 2013). However, in agricultural systems that are major sources of anthropogenic N loss, N is commonly not a major concern because of excessive amendment and ensured availability of N from synthetic N fertilizers and organic manure. Therefore, in several regions, N may not limit the yield because of higher carbon fertilization under elevated CO_2_ conditions, which improves the N and water use efficiencies (Ueyama et al., 2013). If some copping systems can increase yields because of higher carbon fertilization, this could then decrease the excessive availability of soil N than required by plants, ultimately potentially reducing N losses.

## Description of N Cycle

### Ammonification

The second step of N mineralization is referred to as ammonification where reduced organic N (NH_2_) is converted into gaseous ammonia or reduced inorganic (NH_4_^+^) as end product. Various species of microbes, including bacteria and archaea, are involved in ammonification to derive metabolic energy by oxidation of organic N to NH_4_^+^. NH_4_^+^ is the readily available form of N for assimilation and incorporation into amino acids or other metabolic processes. If NH_4_^+^-N is not readily taken up or existing in excess than the metabolic requirements of microorganisms, it will be lost to the environment (Rehman et al., 2016). The rice plant preferably uptakes the NH_4_^+^ form of N rather than NO_3_^–^ (Coskun et al., 2017a). Organic s usage in aerobic rice systems is one the best N management measures to increase NUE (Awan et al., 2014), where the N derived from organic sources is converted to NH_4_^+^ by biological N mineralization. The rate of ammonification in aerobic rice systems is less than that of other agricultural field crops with higher O_2_ depletion during flooding conditions (Belder et al., 2005).

### Nitrification

Nitrification is the conversion of NH_4_^+^ to NO_3_^–^ (Barth et al., 2020) *via* nitrite (NO_2_^–^) by biological oxidation (Black et al., 2019). In rice soils, NH_4_^+^ is readily converted to NO_3_^–^, which accumulates in the soil solution to make its concentrations higher. Conversion of NH_4_^+^ ions to NO_3_^–^ *via* NO_2_^–^ specifies the movement of N through negatively charged soil particles that determines the fate of N in the soil (Skiba et al., 2011). Generally, the nitrification process is divided into two pathways, In the first step, also termed as autotrophic nitrification, autotrophic nitrifiers obtain their required energy by oxidizing NH_4_^+^ to NO_2_^–^ (Jia and Conrad, 2009; Li et al., 2018c). In the second step, also known as heterotrophic nitrification, heterotrophs convert the inorganic and organic N forms (NO_2_^–^) to NO_3_^–^ without gaining any required energy. However, the share of autotrophic nitrification is larger than that of heterotrophic nitrification in N cycle (Hayatsu et al., 2017). The first step is usually performed by AOB, but this operation can also be performed by AOA (He et al., 2018). NO_3_^–^-N is more likely than NH_4_^+^ to move to plant roots *via* mass flow, thereby leaching down from the root zone during soil flooding conditions if not readily taken up or being lost because of denitrification during dry conditions of soil (Farquharson, 2016).

**FIGURE 1:**
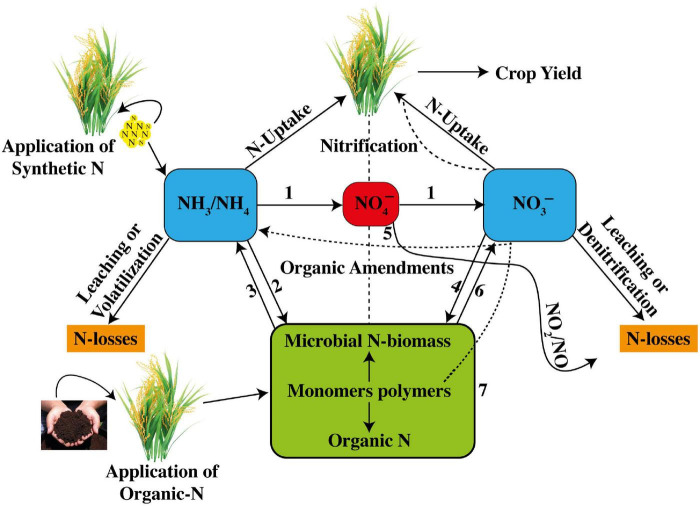
Internal soil N-cycling is comprised of nitrification, ammonification, NH_4_^+^-immobilization, NO_3_^–^-immobilization, dissimilatory NO_3_^–^ reduction to NH_3_ (DNRA), heterotrophic nitrification, and plant N uptake (taking reference of Norton and Ouyang, 2019).

Recent advances in soil research have shown that AOA abundance is higher than that of AOB, but that AOB contribution in nutrification is more than that of AOA in rice soils (Banning et al., 2015). Nonetheless, AOB and AOA communities increased greatly in the rice rhizosphere after the application of a synthetic urea fertilizer (Liu X. et al., 2016; Liu Y. et al., 2016). The process of nitrification either with AOB or AOA is shown below in the reaction form, whereas a general complete description of N cycle internally in soil with all major steps, viz. (1) nitrification, (2) ammonification, (3) NH_4_^+^ immobilization, (4) NO_3_^–^ immobilization, (5) dissimilatory NO_3_^–^ reduction to NH_3_ (DNRA) (Palacin-Lizarbe et al., 2019), (6) heterotrophic nitrification, and (7) monomers uptake by plants, is shown in Figure 1. The general description of N cycle involves how a set of biogeochemical processes converts inorganic and organic N into various forms consecutively from the atmosphere to the soil to living organisms and then back to the atmosphere (Figure 1). N undergoes various procedures of transformations aiming to keep a balance in the ecosystem. N transformations help plants to synthesize chlorophyll from N compounds. During N cycle, ammonification occurs during which bacteria assist in the decomposition process of plants and animal matters, which indirectly facilitates in cleaning up the environment.


NH+4+1.5O2→NO2-+H2O+2H+

$${\text{NO}}_{2}^{-}+0.5{\text{O}}_{2}\to {\text{NO}}_{3}^{-}$$

Latterly, certain species of *Nitrospira* bacteria have been observed in different paddy soils to mediate the entire two-step reaction of NH_4_^+^ to NO_3_^–^ in an organism.


NH+4+2O2→NO3-+H2O+2H+

During the second step of nitrification, NO_2_^–^-oxidation is switched by nitrite-oxidizing bacteria (NOB) belonging to the genera *Nitrobacter*, *Nitrospina, Nitrococcus*, and *Nitrospira* (Pajares and Bohannan, 2016). Certain species of AOA, AOB, NOB, and Comammox (abbreviation of complete ammonia, and the name ascribed to a group of microorganisms that convert NH_4_^+^ into NO_2_^–^ and then into NO_3_^–^ *via* the complete process of nitrification) microorganisms avail their required energy from nitrification pathways by oxidizing inorganic N compounds as they are chemolithoautotrophs (Kuypers et al., 2018). Chemolithoautotrophic species of archaea and bacteria are the dominant microorganisms during nitrification in aerobic rice systems. These microbes are actively dependent on oxidation of NH_4_^+^ and/or NO_2_^–^ for the required energy for their proper growth. Table 1 presents the information about multiple archaeal and bacterial strains and their ecophysiological roles in N cycle.

**TABLE 1:** Details of some archaeal and bacterial strains, their phylum names, and ecophysiological activities.

Domain and genus names	Activity comments	Physiological mode
**Archaea**		
*Methanococcus*	Methanogenesis (*CO*_2_ + 4*H*_2_→*CH*_4_ + 2*H*_2_*O*)	Chemoautotrophic
*Methanospirillum*	Methanogenesis (*CO*_2_ + 4*H*_2_→*CH*_4_ + 2*H*_2_*O*)	Chemoautotrophic
*Halobacterium*	Usually grow in highly saturated salt solutions and extreme halophilic	Photoheterotrophic
*Natronococcus*	Usually grow in highly saturated salt solutions and extreme halophilic	Photoheterotrophic
*Thermoplasma*	Normally grows at 1–4 pH, requires temperature 33–67°C and thermoacidophile	Chemoautotrophic
*Pyrolobus*	Can grow under extreme high temperature ranges up to 113°C and extreme thermophilic	Chemoautotrophic
**Bacteria**		
*Rhizobium*	Plant symbionts, fix N_2_	Chemoheterotrophic
*Frankia*	Plant symbionts, fix N_2_	Chemoheterotrophic
*Erwinia*	Plant pathogens	Chemoheterotrophic
*Agrobacterium*	Plant pathogens	Chemoheterotrophic
*Pseudomonas syringae*	Plant pathogens	Chemoheterotrophic
*Rhodopseudomonas*	Anaerobic phototrophic; are purple non-sulfur and purple sulfur groups	Photoautotrophic, or Chemoheterotrophic
*Chromatium*	Anaerobic phototrophic; are purple non-sulfur and purple sulfur groups	Photoautotrophic, or Chemoheterotrophic
*Chlorobium*	Anaerobic phototrophic; green sulfur group	Photoautotrophic
*Anabaena*	Produce oxygen	Photoautotrophic
*Nostoc*	Produce oxygen	Photoautotrophic
*Prochloron*	Produce oxygen	Photoautotrophic
*Desulfovibrio*	Reduce sulfate	Chemoheterotrophic
*Desulfomonas*	Reduce sulfate	Chemoheterotrophic
*Stigmatella*	Form spore colonies; myxobacteria	Chemoheterotrophic
*Chondromyces*	Form spore colonies; myxobacteria	Chemoheterotrophic
*Streptomyces*	Produce antibodies	Chemoheterotrophic
*Nitrosomonas*	Oxidize N and CH_4_	Chemoautotrophic
*Nitrobacter*	Oxidize N and CH_4_	Chemoautotrophic
*Methylomonas*	Oxidize N and CH_4_	Chemoautotrophic
*Spirochaeta*	Thin, long, and spiral; few are pathogenic	Chemoheterotrophic
*Treponema*	Thin, long, and spiral; few are pathogenic	Chemoheterotrophic
*Bacillus*	Form endospores aerobically	Chemoheterotrophic
*Escherichia*	Enteric and model organism	Chemoheterotrophic

### Denitrification

The microbial respiratory reduction process, in which the NO_3_^–^ and NO_2_^–^ forms of N are converted into gaseous forms of N like NO, N_2_O, and N_2_, is shown below in the reaction form. Reaction 1 corresponds to complete steps of reducing NO_3_^–^ to N_2_ and showcases the intermediate products, whereas reaction 2 represents the complete redox reaction of denitrification showing reduction to end product. Denitrification is primarily mediated by denitrifying microbes, such as *Pseudomonas, Thiobacillus, Bacillus*, and *Alcaligenes*. The process of denitrification involves both heterotrophic (*Paracoccus denitrificans* and various *Pseudomonas)* and autotrophic denitrifiers (*Thiobacillus denitrificans)*. Under flooded conditions, oxides of N serve as alternate electron acceptors to oxygen. Gaseous N_2_O can also serve as an alternative electron acceptor, but non-denitrifiers have the ability to reduce gaseous N_2_O (Bueno et al., 2015). Denitrification ability is infrequently dispersed among various groups of bacteria and archaea based on their taxonomic features (Nishizawa et al., 2013). Reduction of NO_3_^–^ to NO_2_^–^ is mediated by the nitrate reductase enzyme, and conversion of NO_2_^–^ to NO is mediated by nitrite reductase enzyme, while its reduction to N_2_O is conducted in the presence of nitric oxide reductase.


(1) $$NO_{3}^{-}\to NO_{2}^{-}\to NO+N_{2}O\to N_{2}$$

(2) $$2NO_{3}^{-}+10e^{-}+12H^{+}\to N_{2}+6H_{2}O$$

Culture-based findings have shown that soil denitrifying bacteria population in rice soil ranges between 10^3^ g^–1^ and 10^5^ g^–1^ but how diverse they are is still unknown (Ishii et al., 2011; Maeda et al., 2011). Recently, microorganisms involved in nitrification in rice soils have been identified more clearly by crossing functional measures, such as stable isotope probing (SIP) (Yoshida et al., 2012), denitrification functional gene quantification, and functional single cell (FSC) isolation processes. In SIP analysis, succinate is used as an electron donor for denitrification process, which enhances denitrification than fermentation, and DNRA (Albina et al., 2019). A clone library analysis by closely full-length 16S rRNA gene sequencing has demonstrated that *Burkholderiales*, *Rhodocyclales*, and novel *Betaproteobacteria*, intimately related to *Rhodocyclales*, were prevalent in clones obtained, an SIP analysis has revealed that bacteria belonging to *Rhodocyclales* and *Burkholderiales* with closely shared features were dominant members of a ^13^C-succinate-assimilating community (Saito et al., 2008), and a comparative 16S rRNA gene analysis depicted that bacteria belonging to the genera *Herbaspirillum* and *Rhodocyclales* were abundant in rice soils under denitrification-prompting environments (Ishii et al., 2009).

## Volatilization and Leaching

Inorganic N in aerobic rice systems is lost under AWD conditions because of leaching and volatilization. On acidic soils, ammonia volatilization is usually insignificant but under alkaline or calcareous soil conditions, N loss may occur (Ishii et al., 2011). Temperature, pH, and CO_2_ concentration are the major factors influencing ammonia volatilization (Buresh et al., 2008). Ammonium ions have the ability to be adsorbed into soil clay particles, contrary to NO_3_^–^ and NO_2_^–^, which can easily be transported into the soil-water environment and, subsequently, can lead to eutrophication (Buresh et al., 2008). Therefore, during flooding conditions in aerobic rice systems, NO_3_^–^ and NO_2_^–^ are lost because of leaching. Compared with lowland rice systems, however, only a small proportion of NO_3_^–^ is leached down because of strong denitrification pathways. Groundwater samples from a flooded rice field were extracted in Japan, and it was observed that a 0.9% proportion of the samples were contaminated with NO_3_^–^ at ≤10 mg-N L^–1^, and that nearly a 36% proportion of the samples were contaminated with >10 mg-N L^–1^ (Kumazawa, 2002). Moreover, it was concluded that the concentration of NO_3_^–^ in groundwater reached or even exceeded the unacceptable level. An overview representation of ammonification, nitrification, and denitrification of N cycle in rice soils is shown in Figure 1.

## Ecophysiology and Relative Abundance of Ammonia-Oxidizing Archaea and Ammonia-Oxidizing Bacteria

Ammonia-oxidizing bacteria are terrestrial microbes, and they consist of two genera, *Nitrosospira* and *Nitrosomonas*; each of them is comprised of not less than four sub-clusters. *Nitrosospira* spp. are generally found in soil environments, whereas *Nitrosomonas* spp. are widespread in N-rich systems like sewage areas and lakes (Wang et al., 2010; Ke and Lu, 2012). It has been found that the habitat difference for the abundance of AOB species is associated with their physiological characteristics (Norton, 2014). *Nistrosomonas* spp. are termed as r-strategists with a comparatively fast growth rates but low substrate affinities, whereas *Nitrosospira* spp. are considered as k-strategists (Li et al., 2007) with relatively slow and decreased growth rates but high substrate affinities, which can decrease the pollutants’ concentration to low levels. More importantly, K-strategists can also play crucial roles in the biodegradation process of fractious organic pollutants. Fluctuations in substrate availability favor the potential activities of *Nitrosospira* spp., whereas *Nitrosomonas* spp. are better suited under soil conditions with high substrate availability.

All currently known AOA species belong to the phylum Thaumarchaeota in the domain Archaea. AOA exhibit the diagnostic archaeal ammonia monooxygenase (*amoA*) large subunit gene, which encodes the alpha subunit of ammonia monooxygenase and helps to produce the lipid biomarker thaumarchaeol. The *amoA* gene has been widely exploited and utilized as a molecular biomarker to study AOA and AOB distributions and dispersion in various environments, and it is used as a marker of the nitrification process. Ammonia monooxygenase (AMO), a membrane-bound enzyme, is considered as an important functional protein for ammonia oxidation and can oxidize NH_4_^+^ in AOA and AOB. Because of its capacity to encode the active site of ammonia monooxygenase, the *amoA* gene can oxidize ammonia and generate energy for the successive oxidation reaction. The distribution of the archaeal *amoA* gene has shown that the abundance and diversity of archaeal communities associated with soil vary with soil depth and conditions, such as pH, oxygen level, moisture content, salt concentration, and availability of NH_4_^+^ ions (Zhang et al., 2011; Sintes et al., 2013). The subclassification of AOA into the phylum *Thaumarchaeota* forms a separate group of AOA species showing their better adaptation and optimum activities under decreased ammonia concentration in soil. Moreover, an aerobic and mesophilic ammonia-oxidizing archaeon named *Nitrososphaera viennensis* has been found, which utilizes different trophic modes to gain energy for optimum growth (Beam et al., 2014; Stieglmeier et al., 2014). The ecological importance of AOA is still unclear under different environmental conditions (Shen et al., 2012; Huang et al., 2016), but under marine environments they are believed to be dominant, sharing 70% of total oxidized ammonia (Pitcher et al., 2011). There remain many confusions and uncertainties in findings of previous studies on whether AOA are more important in ammonia oxidation (Pitcher et al., 2011; Zhang et al., 2012) than AOB (Shen et al., 2012). However, it has been indicated that AOA abundance was higher at low NH_4_^+^ concentrations, and that high NH_4_^+^ availability favors AOB community abundance (Xia et al., 2011; Pester et al., 2012).

Ammonia-oxidizing archaea and AOB are the major ammonia oxidizers that contribute to ammonia oxidation in agricultural soils, but their relative abundance and share in ammonia oxidation are still challenging to predict (Zhao et al., 2019, 2020), because their abundance and activities are affected under contrasting soil conditions (Prosser and Nicol, 2012). In rice soil usually fertilized with inorganic fertilizers, the top soil layer was found abundant with 80 times more *amoA* (described in later part) copies, which can rise up to 700 times at a 30-cm soil depth (Leininger et al., 2006; Bertagnolli et al., 2016; Han et al., 2018). The ecospecies of NO_2_^+^ and NH_4_^+^ oxidizers in soil govern the rate of nitrification providing substrate excess are ensured (Taylor et al., 2012). Soil microbes, depending on soil conditions, show their potential receptiveness to the addition of synthetic N fertilizers because of temporary increase in substrate availability. A positive correlation between AOB abundance and nitrification rates was observed under high NH_4_^+^ concentration, but there was no or slight correlation for AOA (Ouyang et al., 2016). AOA community abundance showed a strong positive correlation with nitrification rates under acidic soil conditions (Sterngren et al., 2015), because acidic soil with no addition of synthetic N fertilizers reveals lower nitrification rates (Marusenko et al., 2013, 2015). However, recently, it was noticed that synthetic N fertilizers, when added to soil, had a positive and strong correlation with abundance of soil microbes (Ouyang et al., 2018).

## Pathways of Ammonia Oxidation in Ammonia-Oxidizing Archaea and Ammonia-Oxidizing Bacteria

### Ammonia-Oxidizing Bacteria

**FIGURE 2:**
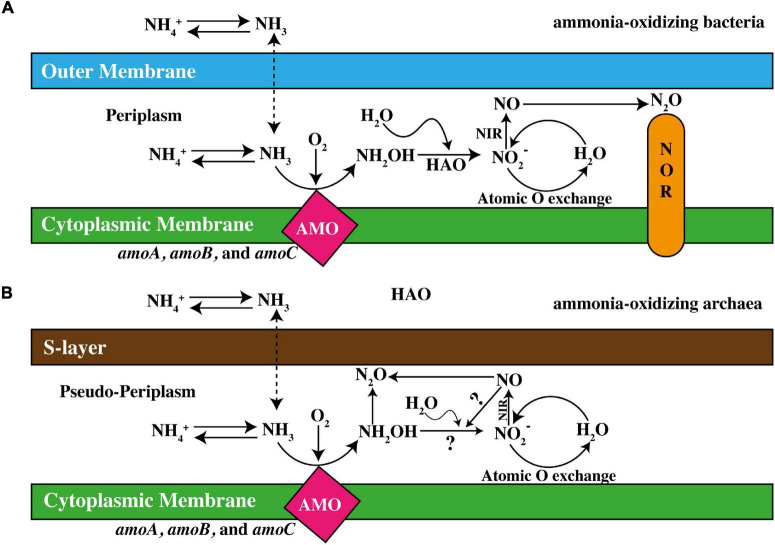
Demonstration of ammonia-oxidation in **(A)** ammonia-oxidizing bacteria (AOB) and **(B)** ammonia-oxidizing archaea (AOA). HAO, hydroxylamine dehydrogenase; NIR, nitrite reductase; NOR, nitric oxide reductase (adapted from Yin et al., 2018).

The rate-limiting reaction in nitrification is ammonia oxidation, which is considered as the main contributor in balancing the relative proportions of NH_4_^+^ and NO_3_^–^ (Urakawa et al., 2006). Microbes responsible for this process, including AOA, AOB, and Comammox, have an enzyme named AMO (Koch et al., 2019), which is the key enzyme in oxidation of NH_4_^+^ (Simon and Klotz, 2013). It catalyzes the conversion of ammonia into hydroxylamine (NH_2_OH, then converted into ${\text{NO}}_{2}^{-}$ by hydroxylamine oxide reductase) (Vajrala et al., 2013). This membrane-bound enzyme is composed of three subunits, produced, and coded by the genes *amoA*, *amoB*, and *amoC* that are arranged as the *amoCAB* operon (Figure 2). Two polypeptide subunits of AMO are coded for by adjacent genes, *amoA* and *amoB*, which are anteceded by a third gene, *amoC*. *amoCAB* operon clusters are present in the form of multiple copies in nitrifying bacteria of the β-subdivision. Considering the homology of *amoA* and *amoC* with other monooxygenases and hydrophobicity projections, they are supposed to the intrinsic part of membrane proteins, whereas *amoB* seemingly accompanies a copper-catalyzed active site (Balasubramanian et al., 2010). Later, the AMO-based end product NH_2_OH is converted into ${\text{NO}}_{2}^{-}$ by the hydroxylamine oxidoreductase (HAO) enzyme (Bollmann et al., 2013; French and Bollmann, 2015) presented in Figure 2. In contrast to AMO, HAO contains ferrous heme P460 in the active site; therefore, it has no susceptibility to inhibition by copper chelators. However, the NH_2_OH obligate intermediate model was postulated which demonstrated that NO is an active and essential intermediate product during the oxidation of NH_2_OH to ${\text{NO}}_{2}^{-}$ in ammonia-oxidizing archaea rather than participating directly in the oxidation of NH_3_ to NH_2_OH. This phenomenon subjected towards the introduction of “NH_2_OH/NO obligate model”. Therefore, NO would not be considered as a byproduct of NH_2_OH oxidation but is preferably required in producing NO_2_ (Liu et al., 2017). This, however, implicates the presence of an anonymous enzyme leading that conversion (Caranto and Lancaster, 2017).

### Ammonia-Oxidizing Archaea

Ammonia oxidation pathways conducted by AOA is less understood (Schleper and Nicol, 2010) in comparison to AOB, but genomic analyses have shown that AOA are occupied with well-defined homologs of genes encoding three subunits of AMO (Stahl and de la Torre, 2012; Kerou et al., 2016). Additionally, the AMO in AOA is accompanying a gene named *amoX* and potentially encodes a fourth AMO subunit (Bartossek et al., 2012). Archaea, are devoid of the active bacterial *amoB* site, which intimates different ammonia oxidation pathways (Tolar et al., 2017). Possibly, another active site in the *amoX* or *amoC* subunit may be responsible for the AMO activity in archaea, the earlier probably less likely due to its transmembrane prophecy (Li et al., 2018b). Even with the nonexistence of HAO homologs, intermediate production and consumption of NH_2_OH exist during archaeal ammonia oxidation, (Kozlowski et al., 2016; Sauder et al., 2018), as shown in Figure 2. Alternatively, various oxidoreductase enzymes have been found contributing in bacterial HAO as P460, and due to their cytoplasmic nature owing no signal peptide for periplasmic secretions such as of energy-efficiency concerns, NH_2_OH production take place in the periplasm (Lawton et al., 2013).

A functionally multifaceted cofactor is found in all archaeal genomes assisting in various low-potential 2-electron redox reactions and could be responsible for HAO activities (Zhalnina et al., 2014). For proper transport of required electrons in archaeal NH_2_OH oxidation, production of NO is probably necessary (Kozlowski et al., 2016; Hink et al., 2018), which is accomplished through a nitrite reductase enzyme (Spang et al., 2012). A schematic illustration of ammonia-oxidation pathways in AOA and AOB is given in Figure 2.

## Interactive Mechanisms of N Cycle, Plant N Uptake, and Microbial Activities

Plant N uptake preference for NH_4_^+^ or NO_3_^–^ could employ varying effects on soil microbes (Thion et al., 2016; Li et al., 2017). Generally, the N-transformation in soil is stimulated by the release of carbon by plants into the rhizosphere either as root exudates or direct release to mycorrhizal communities (Meier et al., 2017). Interactive processes in the rhizosphere reduce the rate of nitrification by microbial assimilation of NH_4_^+^ (Hawkes et al., 2007). Nitrification can be inhibited by nitrification inhibitors (NIs) either released by roots or added artificially (Sun et al., 2016; Coskun et al., 2017b).

Pure cultures of *Nitrosomonas* spp. have been noticed with weak competition for NH_4_^+^ uptake relative to heterotrophic bacteria spp. (Laanbroek and Bär-Gilissen, 2002). Rice grown under different soil conditions has shown very high nitrification rates than microbial NH_4_^+^ assimilation, specifying that soil nitrifiers are powerful and responsive microbes for NH_4_^+^ (Bottomley et al., 2012; Inselsbacher and Näsholm, 2012). Soil microbes having a heterotrophic mode of activities can also assimilate NO_3_^–^ when the soil contains high OM and carbon contents. The competition for NH_4_^+^ is determined by balance between soil carbon and NH_4_^+^ availability, whereas interactions between different soil microbial communities involved in the food web of soil also bridge several reactions in N cycle (Jiang et al., 2014; Trap et al., 2015). Interestingly, it has been found that the community structural composition of AOB entirely got changed because of the presence of bacterivorous nematodes (Xiao et al., 2010). Moreover, the presence of bacterivorous nematodes considerably reduces AOB abundance but encourages AOA community abundance (Zhu et al., 2018). In a study conducted on rice systems grown under wastewater application conditions have shown that *Nitrospira* spp. are consumed by bacterial predators (Zhang et al., 2019b). Complex interactions among soil, plants, and microbes involved in the soil food web are indispensable to nutrient cycling processes, such as soil organic and inorganic N. In the root zone within a soil environment, the interaction between microbes and the root system implicates a range of microbial activities that contribute to C and N dispersal through diffusion and mass flow into the soil composition.

## Factors Affecting the Activities of Ammonia Oxidizers

### Changes in NH_4_^+^ Concentration

Nitrogen, in the form NH_4_^+^, functions as the common substrate of AOA and AOB; therefore, ammonia concentration in soil influences the growth and activity of nitrifying microbes. AOA are highly responsive to NH_4_^+^ compared to AOB (Chen et al., 2017), consequently making low inhibitory level for AOA (Yin et al., 2018). By increasing NH_4_^+^ concentration, the *amoA* gene of AOA was decreased, which stipulated that low NH_4_^+^ concentration favors the growth and activity of AOA (Sauder et al., 2012) and vice versa for AOB (He et al., 2018; Wan et al., 2019). It has been observed that the per cell rates for NH_4_^+^-oxidation and the half-saturation constant for AOA activities were significantly lower than those for AOB (Ouyang et al., 2017). For example, an increase of 100 mg/L in NH_4_^+^ concentration did not produce any significant differences in AOB abundance, but AOA abundance was reduced (Bellucci et al., 2011). In summary, synthetic N fertilizers and high availability of NH_4_^+^ promptly encourage AOB community abundance and activities, whereas AOA activities, abundance, and their response to NH_4_^+^ were not affected by high amendment of synthetic N fertilizers and availability of the NH_4_^+^ substrate (Wang et al., 2022). AOB communities dominantly contribute by their roles in nitrification pathways under nonlimiting NH_4_^+^ whereas AOA dominate nitrification at low NH_4_^+^ concentration in the soil. Understanding and interpretation of different response mechanisms of AOA and AOB to the NH_4_^+^ substrate can permit ammonia oxidizers to be indulged into the simulation as dynamic elements that drive N-fluxes.

### Changes in Soil pH

Soil pH is affected by factors, such as land use changes, soil burning, and accumulation of acids (Malik et al., 2018). Soil microbial populations and their relative activities in biogeochemical processes are affected by changes in soil pH (Bernhard et al., 2010). Among soil microbial communities, especially ammonia oxidizers, changes in soil pH greatly influence bacterial growth and activities (Zebarth et al., 2015), and depict the relative abundance of soil ammonia oxidizers (Prosser and Nicol, 2012). Soil pH is one of the principal factors commanding the rates and end-product accumulations of nitrification (Kyveryga and Blackmer, 2018). Ammonia and nitrite oxidation rates are enhanced under neutral or slight alkaline soil conditions, therefore higher N-loss occurs because of accumulation of NO_3_^–^. Increase in acidification of arable soil encourages high rate of nitrification and leaching of NO_3_^–^ (Schroder et al., 2011). AOB cultures showed no tolerance to acidic conditions, and their rates of nitrification were fiercely decreased with decrease in soil pH (Zhang et al., 2017). Under application of synthetic N-fertilizers, such as urea substrates, the heterotrophic mode of nitrification has been observed under acidic soil conditions (Burton and Prosser, 2001). Ammonia oxidation in acidic soil *via* AOA has been under great consideration since the actual roles of AOA in N cycle have been disclosed, but the literature is still ambiguous with high uncertainties (Afzal et al., 2019). However, the low-pH soil has low intensity of nitrification relative to high pH soil (Shen et al., 2012) where ammonia availability decides the pH of soil (Lu et al., 2012). Moreover, the presence of toxic and heavy metals in soil makes it less fertile, thereby impacting the activity and growth of AOA and AOB. This can be attributed to soil microbes with high tolerance to low-pH soil, and microbes with no tolerance to high acidity are only operative in basic soils (Shen et al., 2012). Recently, it was confirmed that the most active and abundant ammonia-oxidizing microbes are AOA, although AOB were also found but with different response mechanisms (Huang et al., 2016). Physiological and microcosm studies have shown the mechanisms by which changes in soil pH impact the abundance, activities, and growth of microbial functional groups (Verhamme et al., 2011).

### Changes in Soil Temperature

Soil microbial activities are highly reactive to modulation of soil temperature (Naher et al., 2019). Generally, microbial growth and activities are increased under high temperature conditions at a typical temperature range (Heinze et al., 2016). The responsiveness and preferential depletion in the mineral soil of mineral-associated OM under warming is highly dependent on the availability and type of the substrate (Hill et al., 2015). The fate and quantity of available N to the rice plant is determined by soil temperature (Thangarajan et al., 2015). The biological transformation of N is greatly influenced by soil temperature (Daebeler et al., 2018), as nitrifiers are highly responsive to changes in temperature (Osborne et al., 2016) where the rate of nitrification also fluctuates with variations in temperature (Ramanathan et al., 2017).

The nitrification response to varying soil temperature depicted that the optimum temperature required for nitrification is environment-specific (Taylor et al., 2017). Across the range of global ecosystems, the community abundance and diversity of AOB were correspondent with temperature change (Wessén et al., 2011); however, the soil with relatively high dominance of AOA has been observed under the optimum temperature range required to conduct nitrification pathways (Alves et al., 2019). AOB had an optimum soil temperature range of 25–30°C (Hayatsu et al., 2017), whereas a type of soil with AOA dominance was noticed in a winter temperature range in Arctic regions (Alves et al., 2013), which evidenced that AOA communities with high tolerance to acidity are well-adapted to low temperature conditions (Herbold et al., 2017). In contrast, a different study has reported that archaeal population and archaeal ammonia oxidation activities were increased under warmer soil conditions (Tourna et al., 2008). In high-altitude regions, the prevalence of cold periods influences the yearly mineralization of N (Amoo and Babalola, 2017). Some proportions of microorganism communities remained active in unfrozen water films in high-altitude regions to keep a satisfactory level of microbial activity (Schütt et al., 2014). However, it has been noticed that intensive microbial activities stop when soil temperature becomes 5°C (Nottingham et al., 2019). In contrast, optimum activities of nitrifying bacteria and archaea (Santoro and Casciotti, 2011) were observed in the temperature range of 4–8°C (Schütt, 2015). A typical temperature range of 20–36°C for optimized activities of ammonia oxidizers has already been demonstrated (Thangarajan et al., 2015), but for nitrifiers, enhanced activities were noticed specifically at 25°C (Sahrawat, 2008).

The occurrence of soil biological processes (pathways of N-cycle) and climate change are closely correlated with each other in many aspects, as climate variability causes prevalence of biotic and abiotic stresses with adverse impacts on biological processes (Cheng et al., 2015). It has been observed in several climatic regions with high temperature that stress increased the nitrification rates even by up to 50°C (Ihara et al., 2014). The synchronization between interactions of OM and temperature response function may impact the N mineralization process (Takriti et al., 2018). Moreover, it has been observed that temperature impacts the biological activities in N cycle differently compared to inorganic and organic N amendments (Amoo and Babalola, 2017).

### Changes in Oxygen Levels

Nitrification requires oxygen as a necessary substrate, and different responses of soil microbes to oxygen (AOA > AOB) considerably impact nitrification, which also changes with fluctuations in soil available ammonium, water, and pH. Under flooded conditions, the high oxygen response of AOA makes them more competitive over AOB (Sheng et al., 2016). Higher abundance of AOA was seen under hypoxia and low dissolved oxygen concentrations (Zhalnina et al., 2012), which predicted the simultaneous occurrence of nitrification and denitrification processes (Lian et al., 2014). It has also predicted that inhibition of nitrite-oxidizing activities may be conducted by the coupled activity of AOA and denitrifying bacteria (Jang et al., 2019), where N may be assimilated through the simultaneous presence of nitrifiers and denitrifiers (Li et al., 2018a). A real-time PCR quantitative analysis showed the coexistence of microbial communities that include AOA, AOB, NOB, and anammox bacteria (anaerobic AOB have a unique mode of metabolic activities and are able to oxidize NH_4_^+^ and reduce NO_2_^–^ or NO_3_^–^ to produce N_2_ gas) under low oxygen (Yapsakli et al., 2011). N assimilation through mutual activities of soil microbes involved in ammonia oxidation and denitrification (anammox bacteria) can efficiently be performed by regulating dissolved oxygen concentration to optimize microbial community abundance.

### Changes in Soil Water Contents

Soil water availability greatly influences microbial activities, as accessibility of water changes the activity of microbial enzymes (Yan et al., 2015). Nitrification rate is influenced by variation in soil moisture contents, which impact the substrate accessibility of oxygen and NH_4_^+^ by diffusion and through direct effects of dehydration under low water potential. N mineralization is also impacted by changes in soil water availability, as it shifts the required substrate availability to microbes by impacting their growth and internal metabolism (Han et al., 2012). Controlled increase in water contents increases nitrification rate, but submergence of water reduces nitrification rate, as flooding leads to limited oxygen supply (Norton and Stark, 2011). During dry conditions, thin water layers between successive soil particles inhibit the movement of substrates to microbes (Yan et al., 2015). Production of NO_3_^–^ and availability of soil water are positively interlinked, because water serves as the medium for substrate transportation (Wang J. et al., 2017). Microbial cell growth, metabolism, and abundance get adversely affected under high available NH_4_^+^ and higher solute concentration in different soil regimes; therefore, microbial activities become limited because of low water potential (Hu et al., 2015). Moreover, the abundance of the *amoA* gene is directly linked with the availability of soil water contents.

### Changes in Organic Matter Contents

Microbial communities responsible for ammonia oxidation are also influenced by changes in soil OM contents (Gao et al., 2016). Generally, AOB are usually recognized as autotrophs, but it is very difficult and still uncertain to distinguish their mode of action either as autotrophic or mixotrophic. It has been found that high OM significantly inhibited the growth of certain AOA strains, i.e., *Nitrosopumilus maritimus* and *Nitrosocaldus yellowstonii* (Rusch, 2013). It has been discovered that increase in OM content encourages the growth of AOA strains having a mixotrophic mode of growth and activity (Qin et al., 2014). Genome sequencing has proven that various AOA strains had carbon utilization mechanisms namely 3-hydroxypropionic acid/4-hydroxybutyric acid cycle named as autotrophic metabolism. Moreover, tricarboxylic acid cycle was named as heterotrophic metabolism, which consequently demonstrated that AOA strains are equipped with both the potential autotrophic and heterotrophic modes of metabolisms (Beman et al., 2008). Comparing the structural composition of soil microbial communities, it has been assessed that there may exist a nexus of metabolic mechanisms in AOA with diversified metabolic characteristics at different carbon levels, eventually depicting different ammonia oxidation capacities of ammonia oxidizers (AOA and AOB).

### Changes in Bioavailability of Nutrients

Microbial activity is reduced under limited availability of required substrates and is a significant feature in nitrification activity. Production of NO_3_^–^ from ammonia oxidation pathways is conducted by either chemoautotrophic or heterotrophic nitrifiers (bacteria, fungi, and archaea) (Pajares and Bohannan, 2016). The contribution of microbes in biological processes can be estimated through the C/N ratio of the required substrates. The bioavailability of carbon is intensified under higher C/N ratio stimulating the growth of microbes (heterotrophs) that inhibit the activities and growth of autotrophs. Substrates required for nitrification can be taken from inorganic and organic sources. N-containing compounds play the role of a substrate, as the N from organic sources has been observed to be used as substrates in N cycle on acidic soils (Zhang et al., 2014).

**FIGURE 3:**
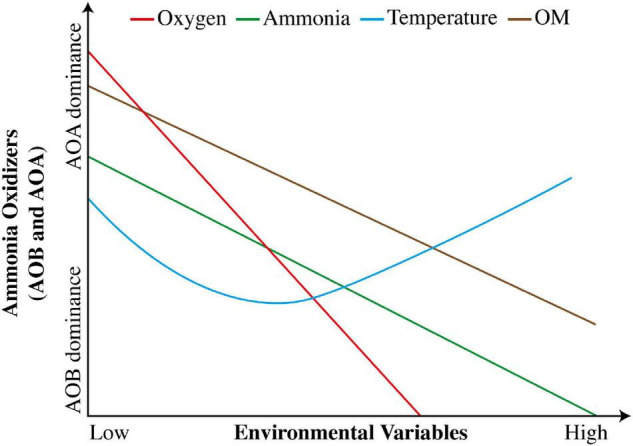
Hypothetical variation in responses of AOA and AOB to changes in environmental factors (adapted from Ouyang et al., 2017).

The substrates required in N cycle determine the type of vegetation in any climatic region (Stein and Klotz, 2016). The growth, abundance, and activities of microbial communities in N cycle are affected by plant population (Bosco et al., 2015). The bioavailability of nutrients has an impact more than other factors, because addition of any required substrate to soil and uncommon changes in soil pH may occur (Zhang et al., 2016). Low NH_4_^+^ level may have concerns with reduction in growth and activities of AOA (Norton and Ouyang, 2019). During N cycle, the concentrations of required NH_4_^+^ substrates vary for AOA and AOB (Leininger et al., 2006). AOA are presumed to work well under low soil fertility and oligotrophic conditions (Liu et al., 2018), whereas AOB work appropriately under opposite conditions (Erguder et al., 2009; Daebeler et al., 2012). Meanwhile, AOB survive better under high concentration of NH_4_^+^, whereas AOA thrive superiorly at low level of NH_4_^+^ (Sterngren et al., 2015). A hypothetical illustration of variations in responses of AOA and AOB to changes in environmental factors is shown in Figure 3.

## Agronomic Management Measures

### Increasing N Use Efficiency and Development of Nitrification Inhibition Assays

Among rice-producing countries, China is the top intensive user of N fertilizers for higher rice production to meet its national food demands. However, the country should have adopted management measures, such as use of controlled-release fertilizers, NIs, N-splitting, basal and deep placement of N fertilizers, and optimizing the rate and timing of fertilizer application to increase NUE (Xia et al., 2017). The application of N fertilizers based on soil requirements can be effective to minimize N-losses due to leaching, volatilization, and runoff in aerobic rice systems, thereby enhancing NUE and reducing environmental hazards. Splitting and coating of total required N along with use of NIs can establish economic benefits and help in meeting food demands under scarce resource availability and selective conditions (Nelson et al., 2014). Soil management practices, such as zero or no tillage, crop rotation, soil mulching, and crop residue amendment, can improve soil health and nutrient availability, resultantly making aerobic rice systems more resilient (Zhang et al., 2015). Moreover, soil and N fertilizer management practices can ensure environmental sustainability with enhanced NUE and sustainable production in aerobic rice system same as the conventional.

#### Inhibition of Nitrification Through Nitrification Inhibitors

Nitrification inhibitors reduce the speed of microbial conversion pathways of NH_4_^+^-N to NO_3_^–^-N, reducing the risk of possible N-losses by leaching or volatilization, thereby increasing NUE (Menéndez et al., 2012). Many synthetic NIs target the ammonia monooxygenase enzyme often acting as a competitive suicide substrate (acetylene) (Ruser and Schulz, 2015). Irrigation management strategies through the AWD technique in aerobic rice systems cause coupled nitrification-denitrification pathways, which are subjected to high N losses. Therefore, to reduce N loss and avoid the negative impacts of nitrification, various Nis can be commercially manufactured and widely used. Most recurrently used commercial NIs include dicyandiamide (DCD), nitrapyrin, and 3,4-dimethylpyrazole phosphate (DMPP) (Suter et al., 2010) that act as metal chelators to bind copper (Cu) on the active site of the *amoB* subunit (Ruser and Schulz, 2015). Urease inhibitors, i.e., N-(n-butyl) thiophosphoric triamide (NBPT), are used to decrease synthetic urea hydrolysis and volatilization. Moreover, nitrapyrin can be used as an alternative *amoB* substrate, consequently producing an end product that irremediably stops ammonia oxidation. It has been evidenced that in a *Nitrosomonas europaea* probe, using either ammonia or hydroxylamine as a substrate, nitrapyrin and DCD were evidenced to deploy the inhibition of AMO, not HAO (Zakir et al., 2008; Pariasca Tanaka et al., 2010). Amazingly, nitrapyrin is equipped with a very weak nitrification inhibition capacity toward AOB (Shen et al., 2013). It has also been found that application of NIs (including urease) significantly reduced the leaching loss of inorganic N by 48%, N_2_O emission by 44%, and NO emission by 24% (Qiao et al., 2015) and that it increased agricultural production by 7.5% and NUE by 12.9% (Abalos et al., 2014; Recio et al., 2018).

The strength of NIs under different soil conditions is impacted by temperature fluctuations and plays a key role for their efficient inhibition activities (Guardia et al., 2018a). For optimum balance between GHG emission and N oxides, improved NUE and crop yield preferably require the use of Nis of organic sources in aerobic rice systems (Recio et al., 2018). Nevertheless, NI use under field conditions may increase environmental pollution because of high potential of NH_3_ emission (Qiao et al., 2015). Many potential NIs have been discovered, but some concerns restrict their widespread application under field conditions; usually, they are equipped with alkynes functional groups potentially hindering ammonia oxidation (Taylor et al., 2015). Acetylene is known as a general NI with a short-chain alkyne group because of its ability to bind the *amoA* subunit instead of *amoB* (Bennett et al., 2016). The nitrous oxide scavenging observed among NO scavengers that showed their receptibility with AOA was proposed to block the necessary electron transport to parallel bacterial HAO (Hu et al., 2013). Mixed application of NIs is suggested to target different levels of ammonia oxidation pathways, which can reduce the possibility of resistance development (Coskun et al., 2017b). Moreover, commercial chemical NIs are not recommended in highly certified and organically produced cropping systems; therefore, organic alternatives for such NIs are necessary to control nitrification (Opoku et al., 2014).

#### Nitrification Inhibition Through Biological Nitrification Inhibitors

Certain plants produce root exudations, which act as NIs when exposed to NH_4_^+^ substrates; this process is termed biological nitrification inhibition (BNI), and compounds involved in this process are known as BNIs (Byrnes et al., 2017; Nuñez et al., 2018). Rice rotation with crops producing BNIs potentially enable cash and catch crops to block the activities of HAO and AMO (Subbarao et al., 2009). Root exudations of BNIs are better and beneficial than synthetic NIs (Tesfamariam et al., 2014), as their impacts are short-term and dependent on soil conditions (Ruser and Schulz, 2015). Notably, methyl 3-(4-hydroxyphenyl) propionate (MHPP) is one of the BNIs that stimulates root branching and increases the potential of N hunting because of a dual kind of functionality (Meena et al., 2014). Some BNIs have been extracted from leguminous crops in tropical regions, i.e., *Sorghum bicolor* has been observed to have nitrification inhibition abilities (Subbarao et al., 2013, 2015). Root exudates containing BNIs are also stable under different soil conditions, and the production of BNIs is mainly restricted to the rhizosphere; hence, the modes of action of these compounds could be more efficient. *Brachiaria humidicola* is a tropical pasture grass and one of the well-understood BNI-containing plant species with capacities to grow under highly acidic soil and humid environmental conditions, and it produces the biochemical BNI compound known as brachialactone. Blending of such leguminous and non-leguminous crops into rice crop rotation may help in increasing the soil capacity to retain N and soil N-pools. Transferring the BNI traits of green manure crops into cereal crops will potentially increase NUE with some trade-offs to production.

#### Nitrification Management Under Changing Climate

**FIGURE 4:**
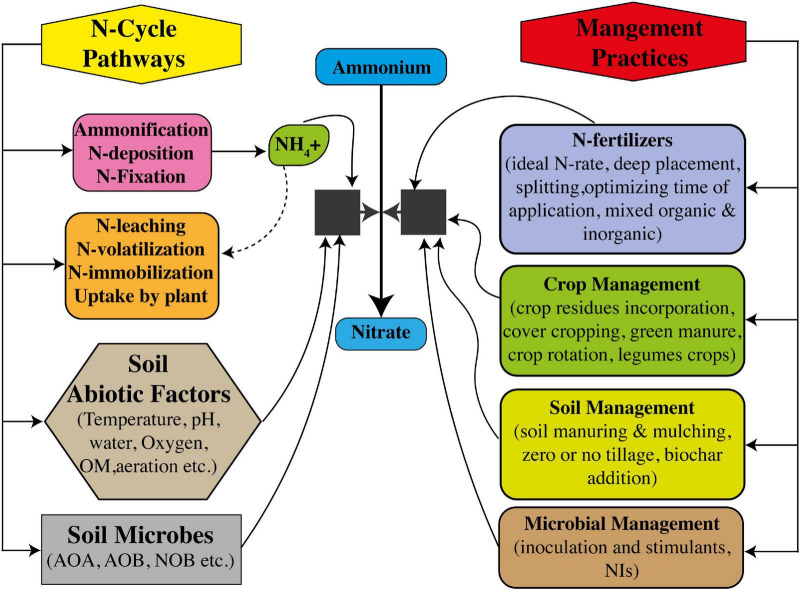
Interactive control of nitrification to increase nitrogen use efficiency (NUE) through soil microbes and management measures of soil, crop, and N fertilizers.

Modern rice production systems require plenty of management measures to reduce N losses under climate changes, which have been going for centuries. The aim of reducing N losses in modern production systems is seemingly difficult considering the technical, management, and socioeconomic consequences. Soil specificity-based management strategies are necessarily required to limit the residence time of N in soil while promoting the maintenance of N retention capacity of soil. Limiting the field fallowing after harvest, use of mulches and cover crops, green manure amendments, and crop rotation practices are beneficial practices to reduce N losses, allowing soil to retain more N (Figure 4). Now, it is well-understood that N-cycle is impacted by climate change, which brings certain changes in nitrification pathways (Robertson et al., 2013), such as changing notification rates and its tendency and subjectivity, toward higher N losses by leaching and volatilization (Li, 2007). As described earlier, nitrification pathways are totally dependent on availability of soil NH_4_^+^ substrates, moisture content, temperature, pH, and textural conditions (He et al., 2019). Moreover, trait-based models have shown that during nitrification, changes in activities and abundance of AOA, AOB, and NOB communities can occur because of climate change (Bouskill et al., 2012; Bowles et al., 2018). However, fertilizer application management strategies, inputting synthetic NIs and BNIs, and management of irrigational water are totally dependent on farmer choice. Therefore, such management should necessarily be applied in modeling research on soil nitrification to recommend modifications on local scales in these practices under climate change.

### Optimizing Time of N Fertilizer Application

Conventionally, application of N fertilizers can be performed before planting or in splits where the quantity can be maintained based on growth stage requirements. The plant N uptake and NUE of different N fertilizers can be enhanced by ensuring the application of N fertilizers as per crop growth stage requirements rather than prior to crop planting (Morris et al., 2018). Splitting of N fertilizers and timing of application should match the specific plant growth stage after proper soil testing where the crop is grown. Side-dressing of N fertilizers is one of the best fertilizer management measures that can increase plant N-uptake and NUE by reducing N_2_O production by 60% (Guardia et al., 2018b). There are vast areas globally where it is more convenient to apply anhydrous NH_3_ during dry soil conditions aiming to retain higher soil N with reduced nitrification rates. Under dry soil conditions, there are limited economic benefits of N fertilizer application; therefore, it is recommended to apply N fertilizers when temperature and moisture contents are in optimum ranges to delay nitrification. Application of Nis along with synthetic N fertilizers in aerobic rice systems under the AWD system to delay nitrification can greatly increase NUE.

### Ensuring Better Assimilation and Utilization of Available N

The availability of N nutrients in both organic forms, such as urea and free amino acids, and inorganic forms like NH_4_^+^ and NO_3_^–^ under different soil conditions greatly differs and impacts plant growth, and physiological, metabolic, and root morphological features. Soil available N can change nitrification rates and NO_3_^–^ accumulation under high competition conditions for N uptake. The N retention capacity of soil and plant roots in organic forms greatly depends on soil properties and plant root morphological characteristics. Paradoxically, when nitrification is happening, low nitrification rates can be observed because of high NO_3_^–^ uptake by plants and microbes (Norton and Stark, 2011). Aiming to increases the direct N uptake in plants (by substantial ammonification) with suppression of nitrification, a wide range of N conservation measures have been developed for agricultural systems. Fundamentally, these essential measures limit N cycling with increase in organic N accumulation in soil. These essential measures include rice crop rotation with cover crops and legumes, synthetic N fertilizer management, and OM incorporation, which support the subsistence of rice plant roots, improve plant N uptake, and decrease N losses (He et al., 2019).

### Modifying and Strengthening N Cycle

Aerobic rice systems with intensive amendments of inorganic N fertilizers share soil N cycling with higher nitrification rates. Generally, NO_3_^–^ is considered as the dominant form of N right after the application of synthetic N-fertilizers. It is well-known that the quantity of soil available N increases after fixation and ammonification depending on N fertilizer source, and rice systems with reduced rates of nitrification are recommended to limit N losses and improve NUE. To ensure higher and sustainable grain yield in aerobic rice systems, it is necessary to ensure the sufficient availability of soil N in either of the preferable plant-available forms, i.e., as NO_3_^–^ or NH_4_^+^, during the crop-growing season (Javed et al., 2022). As a result of OM mineralization, these available forms of mineral N compounds are unremittingly released in soil solution and available to plants. The release of mineral N compounds as with OM turnover, also termed as “N-delivery,” is majorly dependent on the climatic, soil, and cultivation features. Although there were large-scale and diversified research studies conducted in the past, so far, there have been uncertainties in estimation of available mineralization rates under aerobic rice systems. Therefore, fertilizer management strategies are necessary to bring modifications in overall N cycling, which can ensure the availability of preferable form of N for plant uptake.

**FIGURE 5:**
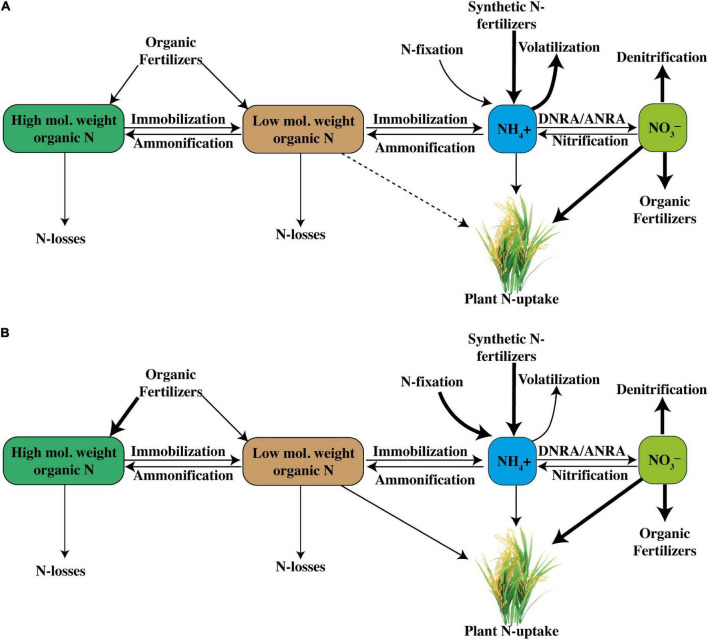
Conjectural comparison of N-cycle under a **(A)** rice system with higher nitrification rates and a **(B)** rice system with relatively low nitrification rates (DNRA, dissimilatory NO_3_^–^ reduction to NH_4_^+^; and ANRA, assimilatory NO_3_^–^ reduction to NH_4_^+^).

Diversification of microbial communities having functions in N cycling can also favor the soil N retaining capacity. For better assimilation of applied N, intensification and modifications in N cycle are necessary with incorporation of high carbon-containing organic fertilizers particularly green manure, biochar, and compost (Paustian et al., 2016). Moreover, direct inoculation of N fixing and ammonification encouraging bacteria in N cycling of intensified aerobic rice systems can also improve the assimilation of soil-amended N (Hu and He, 2018). It has been depicted that use of inorganic N sources makes N cycling easier but at the cost of high N losses due to higher nitrification rates, whereas amendment of organic sources favors N fixation and ammonification and ultimately increases soil N availability for plants. Therefore, a simplified aerobic rice cropping system with less nitrification rate by proper selection of either organic or inorganic N fertilizer sources is necessary to increase the overall NUE (Norton and Ouyang, 2019), as shown in Figures 5A,B.

## Conclusion

### Importance of Aerobic Rice Systems

Globally, rice systems are faced with the challenges of having higher yields with decreased irrigational water. The sustainability of the flooded rice system is being threatened by scarcity of inputs, such as irrigational water, labor, and energy. Aerobic rice systems can be a valid option to sustain future rice yield under scarce freshwater resources through proper agronomic management measures. One of the main benefits of aerobic rice systems over the conventional is reduction in use of resources, such as irrigations with higher water productivity and less labor cost. Low yield in aerobic rice systems suggests the need to undertake necessary measures in the management of N losses and weeds. A big yield gap between aerobic and conventional rice systems can be reduced under favorable conditions if coupled with proper agronomic management practices in minimizing weed invasion and N losses. If desired soil chemical and physical properties are satisfied, timely sowing, proper stand establishment, and higher grain yield can be ensured under aerobic rice systems unlike under the conventional rice system where land preparation is more laborious.

An aerobic rice system is a production setup where rice is grown under well-drained, non-saturated, and non-puddled soil conditions. Resource use efficiency is very high because of less use of irrigational water during land preparation, no transplantation requirements, and less labor costs from sowing to harvesting. Cultivars that are cultivated in aerobic system must have better yield performance traits of lowland conditions and better drought tolerance characteristics of upland conditions with required root traits. Physiological traits in terms of protein and chlorophyll contents, flowering, relative water contents, osmotic adjustments under drought stress, and enzyme activities are the major attributes that play major role in widespread adaptation of aerobic rice systems. Low NUE is one of the major problems for yield decline and adaptation of aerobic rice systems due to changes in soil nutrient status and difference in microbial activities. Aerobic rice systems undertaking agronomic management measures of applied N fertilizers to improve the abundance and activities of microbial communities important in N cycle may lead to sustained grain yield. This will also help in environmental sustainability in the scenario of climate warming by reducing GHG emission, which is an added benefit of this system.

### Key Findings of the Review Article

Nitrogen loss is one of the major issues in both conventional and aerobic rice systems, although in the latter losses are relatively very high. The conventional rice system needs some transformational approaches, such as aerobic rice systems, because of the future projected scarcity of resources. However, the higher rates of N losses in aerobic rice systems threaten the wider adaptation of these transformed rice systems and cause environmental hazards. For widespread adaptation of aerobic rice systems, reducing N losses is necessary by adjustments in microbial communities involved in N cycle *via* deep insights into pathways and their ecophysiology. Therefore, this study was designed to investigate the ecophysiology of soil microbial communities involved in N-cycle, their diversification, abundance, and activities, and how their activities are affected under different soil conditions. This study discusses the recent discovery of AOA, which widens the knowledge of N cycle and shattered the conventional perspectives for the past 10 decades that ammonia oxidation is only controlled by AOB. Hence, the N cycle in aerobic rice systems requires reconsiderations, as some recent novel developments in N cycling have depicted changes in activities and dominance of AOA rather than AOB under different environments. The conjoint, competitive, and inhibitive microbial interactions need further exploration in conventional and aerobic rice systems to increase overall NUE. This study discussed the mechanisms of how highly active ammonia oxidizers interactively impacting N-cycle and plant N uptake. After understanding the interactive pathways, this study narrowed down several adjustive management measures to limit N-losses that may enhance NUE in aerobic rice system, reducing GHG emissions and environmental hazards.

Ammonia is predominantly found in nature and considered as a substrate to produce NO_3_^–^. AOA and AOB are primary ammonia oxidizers that engage in conversion of NH_4_^+^ to NO_3_^–^, which is the most preferable form to be utilized by the rice plant. This article suggests management measures for fertilizers, soils and crops to adjust the functioning of AOA and AOB and better assimilate soil available N. It also discusses measures to control nitrification rates through NIs and BNIs, measures to enhance NH_4_^+^ substrate availability, managing the time, type, and application methods of N-fertilizers, and other measures for ensuring better plant N assimilation and utilization.

### Few Open Questions and Future Thrust

Currently, the most important point to be taken is the biochemistry of archaeal ammonia oxidizers and, therefore, its implications for habitat separation from AOB. The estimation of substrate ranges required for AOA and AOB as well as the involvement of substrate range in losses of N can fundamentally help in the understanding of the impacts of archaeal and bacterial ammonia oxidizers on soil biochemical processes. Another issue currently considered not sufficient is the relationship between AOA and AOB and nitrite oxidizers. The mutual dependency of nitrite oxidizers on AOA and AOB is one of the typical examples of the metabolic association of three functional groups of microbes. Therefore, insufficient knowledge of co-dependence of AOA and AOB on nitrite oxidizers is potentially unfortunate. Moreover, the evaluation of spatial and temporal co-occurrence of these communities under different soil conditions is still unstudied. Further research studies are suggested to conduct several cultivation-based experiments to cultivate AOA and AOB under more strictly controlled conditions. It will be helpful for better understanding of the response mechanisms of AOA and AOB to environmental variability under conditions that will allow AOA and AOB for better utilization of their metabolic functions under natural conditions.

## Author Contributions

MF, ZM, and MU conceived the idea and collected the relevant literature. MF and MU visualized the figures. MF, ZM, MY, MK, SF, SY, and MU helped in writing the original draft. All authors carefully read, revised, and approved the article for submission.

## Conflict of Interest

The authors declare that the research was conducted in the absence of any commercial or financial relationships that could be construed as a potential conflict of interest.

## Publisher’s Note

All claims expressed in this article are solely those of the authors and do not necessarily represent those of their affiliated organizations, or those of the publisher, the editors and the reviewers. Any product that may be evaluated in this article, or claim that may be made by its manufacturer, is not guaranteed or endorsed by the publisher.
